# Failure mode effect and criticality analysis of ultrasound device by classification tracking

**DOI:** 10.1186/s12913-022-07843-4

**Published:** 2022-04-01

**Authors:** Longchen Wang, Bin Li, Bing Hu, Guofeng Shen, Yunxin Zheng, Yuanyi Zheng

**Affiliations:** 1 Department of Ultrasound in Medicine, Shanghai Jiao Tong University Affiliated Sixth People’s Hospital, Shanghai, 200233 China; 2 Department of Biomedical Engineering, Shanghai Jiao Tong University Affiliated Sixth People’s Hospital, Shanghai, 200233 China; 3 School of Biomedical Engineering, Shanghai Jiao Tong University, Shanghai, 200030 China

**Keywords:** Ultrasound device, Failure analysis, Risk assessment, Failure mode

## Abstract

**Background:**

Medical ultrasound device has been more and more widely used in the hospital and Its safety risk is significantly increased when failures occur. However, there is a lack of quantitative risk assessments of different types of failure modes for medical ultrasound device. This study utilizes a failure mode, effect and criticality analysis (FMECA) approach for quantitative risk evaluation of different failure modes for ultrasound devices.

**Methods:**

The 4216 medical ultrasound device failure records at various hospitals were investigated. A failure mode and effect analysis method was developed for the quantitative evaluation of the risks of different failure modes. Visual correlation analysis was conducted for all categories to identify the causes of various failure modes. Based on the severity, occurrence and detectability of the failure causes determined, the risk priority number (RPN) for each failure mode was back-calculated through the obtained tracking diagram.

**Results:**

The failure modes of unclear images, unable to power on and dark shadows on an image had the highest RPNs. One failure mode could be caused by various factors, and the failure location was not necessarily related to the cause of the failure.

**Conclusions:**

This quantitative approach more accurately evaluated the risks of different failure modes, and the results of the corresponding analysis of failure modes and causes could support the rapid determination of the causes of failures in clinical practice.

## Background

Medical errors have always constituted an important problem in medical services. The frequent occurrence of medical errors can seriously threaten the lives and safety of patients and result in substantial economic losses [1, 2]. Research has shown that more than 170,000 people die of medical errors every year, representing the third most common cause of death in the United States [3]. Regarding the use of pacemakers and implantable cardioverters, FDA device malfunction reports revealed that 61 deaths annually were attributed to device malfunction from 1990 to 2002 [4]. The Implant Files report showed that 2 million injuries and 80,000 deaths were associated with medical devices from 2008 to 2017 [5]. As medical devices are widely used in the diagnosis, cure and treatment of disease, adverse medical device events and medical device safety surveillance have attracted widespread attention [6, 7]. A survey revealed that the unexpected failure of medical devices could cause adverse events in radiology departments [8]. The risk of failures of medical devices must be analyzed and assessed, and measures regarding the quality control of devices are essential for ensuring and improving patient safety [9].

With the advantages of cost effectiveness and real-time imaging [10], ultrasound methods, such as transesophageal echocardiography [11], the detection of pulmonary congestion [12], guidance for live biopsy [13], the cannulation of the subclavian vein [14] and the establishment of vascular access in critically ill patients [15], have been widely used in hospitals. However, the risk associated with these methods increases substantially if device failure occurs. Many efforts have been made to analyze the failure of ultrasound devices and adopt efficient safeguard measures. The failure modes and rates of various failures were studied, and routine quality control plans were recommended based on one or several radiological assessments [16, 17]. The causes of transducer failures were further investigated to improve the detection of failures in advance [18–20]. However, there is a lack of quantitative risk assessments of different types of failure modes for medical ultrasound devices. In recent years, failure mode, effect and criticality analysis (FMECA) has been used as a risk assessment approach for medical devices [21, 22]. Conventional FMECA is used to directly score the failure mode [1, 23], and it is not very accurate for risk assessment, as identical failure mode may be induced by different causes. Moreover, due to a lack of engineering experience, physicians cannot determine the causes of failure, which may delay the effective resolution of failures, which could have further serious effects.

To identify the risks of various failure modes and determine the failure causes, we utilized an FMECA approach for the quantitative risk evaluation of different failure modes of ultrasound devices. The 4216 failure records for 2096 sets of ultrasound devices at various hospitals were collected and analyzed. First, the failure modes and causes were marked and classified for every record. Cluster analysis was performed separately for various failure modes and causes, and visual correlations for different categories of failure modes and causes were established. Then, by applying the analysis model, the risk priority number (RPN) for each failure mode was back-calculated based on the severity (S), occurrence (O) and detectability (D) of failure causes. Given that the final effect of failure is directly related to the cause, this quantitative risk assessment method is more accurate than traditional methods. Preventive strategies are discussed for high-risk failure modes.

## Methods

### Data collection and sampling

Maintenance records from 2017 to 2019 at the sampled hospitals were collected. The items that lacked failure descriptions were excluded, and the analysis only included work orders that could be classified based on failures and not those that had other or empty classification results, such as management activities, waiting for spare parts, and preventive maintenance. The failure data were sorted by different persons three times, and failure classification terms were reviewed by ultrasound maintenance engineers with many years of experience. Finally, 4216 valid items were left for analysis, which encompassed 9 brands and 2096 devices. The usage duration of the devices varied from 1 to 18 years, and 75.1% of the usage durations were less than 8 years.

### Ranking criteria

The Delphi expert consultation method was used for the ranking of the severity of failure causes [24]. Fifteen experts, including clinical engineers, ultrasound physicians, original manufacturing engineers and medical device administrators, participated in the analysis. The ranking indicators are summarized in Table 1. The severity of a failure was scored from 1 to 10 based on factors including maintenance efficiency, maintenance cost, impact on safety and impact on diagnosis. As shown in Table 2, the occurrence (O) was quantified through maintenance records. We determined the occurrence ranking using the proportion of failures to reflect the frequency [22]. One failure that occurred out of 4216 cases (1/4216 = 0.0002) was ranked as 2, whereas no failure was ranked as 1. The detectability (D) of failure was divided into five levels according to the difficulty of failure detection, shown in Table 3. The higher the score is, the more difficult the detection of that failure type.

**Table 1:** Ranking criteria of the severity

Score	Severity			
	Maintenance efficiency	Maintenance cost	Impact on safety	Impact on diagnosis
**1** ~ 2	No spare parts need to be replaced, repair time < 4 h	Cost ≤ 10,000 RMB	No impact	No impact on diagnosis
3 ~ 4	Spare parts need to be replaced, repair time < 8 h	10,000RMB < Cost ≤ 50,000RMB	No impact on personnel	Slight impact on diagnosis
**5** ~ 6	Spare parts need to be replaced, repair time < 24 h	50,000RMB < Cost ≤ 200,000RMB	Impact on personnel health	Impact on diagnosis
**7** ~ 8	Spare parts need to be replaced, repair time < 48 h	200,000 RMB < Cost ≤ 1,000,000RMB	Causes injury to the patient	Serious impact on diagnosis
**9** ~ 10	Spare parts need to be replaced, repair time ≥ 48 h	Cost > 1,000,000 RMB	Cause serious injury to the patient	Unable to carry out diagnostic work

1 US dollar≈7 RMB

**Table 2:** Ranking criteria of the occurrence

Score	Occurrence (failure frequency)
1	< 0.02%
2	0.02%
3	≤ 0.2%
4	≤ 1%
5	≤ 2%
6	≤ 4%
7	≤ 6%
8	≤ 8%
9	≤ 10%
10	> 10%

**Table 3:** Ranking criteria of the detectability

Score	Detectability
1 ~ 2	The failure can be determined by phenomenon or visual inspection
3 ~ 4	The operator can simply judge the failure mode
5 ~ 6	Engineers can mostly detect failure modes with the help of professional tools
7 ~ 8	A variety of methods and tools are needed to detect the failure mode
9 ~ 10	Known detection methods are insufficient to detect the failure mode

### Modeling and data analysis

**Fig. 1:**
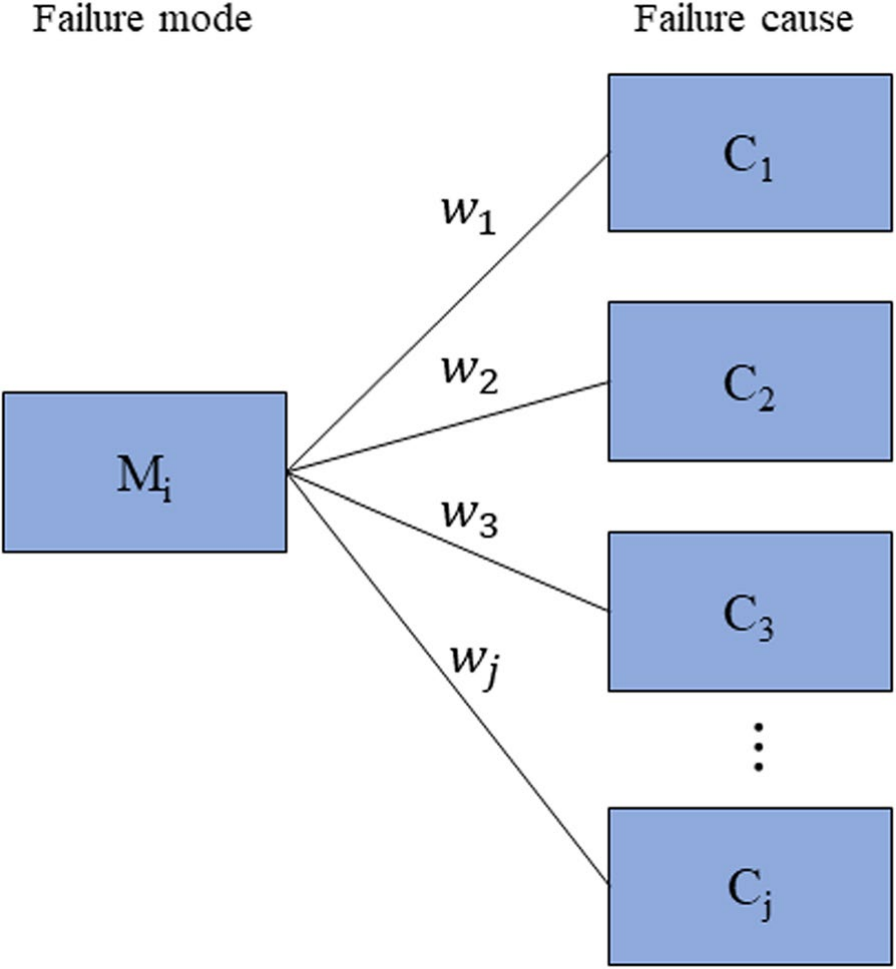
Modeling of the calculation of severity, occurrence and detectability of failure mode

The severity, occurrence and detectability (SOD) of failure causes were scored according to Table 1, Table 2 and Table 3, respectively. Then, the SOD of the failure mode was determined using the model shown in Fig. 1. The severity of the failure mode was calculated using the following equation [25]:

1 $${S}_{Mi}={{\sum }_{j=1}^{k}{w}_{j}S}_{cj}$$

where ${S}_{Mi}$ represents the severity of the failure mode $i$, ${S}_{cj}$ represents the severity of the failure cause $cj$, $k$ represents the type number of potential causes of failure mode $i$, and ${w}_{j}$ represents the proportion of each cause. ${w}_{j}$ is calculated as follows:

2 $${w}_{j}=\frac{{n}_{j}}{{\sum }_{j=1}^{k}{n}_{j}}$$

where n represents the frequent number of each failure cause $cj$. The detectability and occurrence of the failure mode was also determined using the model above. The final RPN for each failure mode was calculated with the equation RPN = S*O*D [26]. The R language package was used for the analysis.

## Results

### Failure classification

**Fig. 2:**
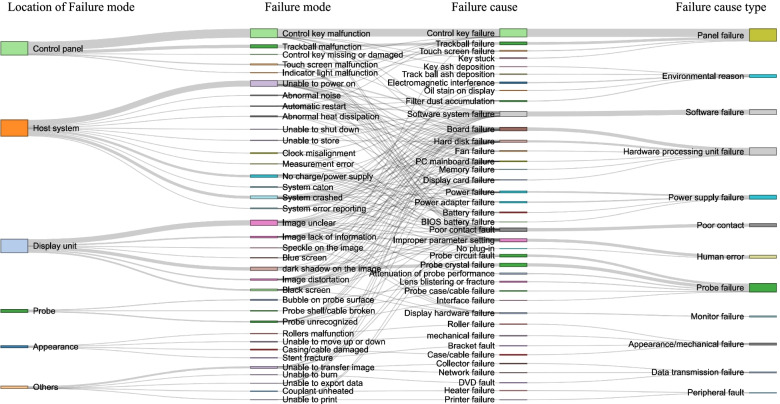
Correspondence between different failure modes of ultrasound devices and their causes

To construct the relationship between failure modes and causes, each repair order was reviewed. As shown in Fig. 2, all failure modes were divided into 36 types, and all the failure causes were divided into 39 types. According to the location in the system, the failure modes were further clustered into 6 categories, namely, the control panel, probe, appearance, display unit, host system and “other”. The control panel category included failures related to the control panel, such as touch screen, trackball and control key issues. The failures of the display unit mainly manifested as unclear images, striped black shadows and black screens. The failures of the host system mainly included not powering on, shutting down and failing to charge. The probe failures were characterized by bubbles on the probe surface, a broken shell or cable and unrecognized probe errors. The appearance failures included roller malfunction, inability to move up/down, casing/cable damage and stent fracture. Other failure modes included being unable to transfer images, being unable to burn, print, or export data and having unheated couplants. The failure causes were further categorized into 12 major cause types, including panel failure, environmental reasons, software failure, hardware processing unit failure, power supply failure, poor contact, human error, probe failure, monitor failure, appearance or mechanical failure, data transmission failure and peripheral fault. Each major cause category included one or more cause types.

Through the relationship between each failure mode and the corresponding cause, the relationship between the location of failure modes and types of failure causes was established. The number of failures in the Sankey diagram is represented by the thickness of the lines. The location of a failure is not necessarily related to the cause of the failure. The phenomenon of being unable to power on the device was related to 13 types of causes, indicating that the failure could not be assessed very easily.

**Fig. 3:**
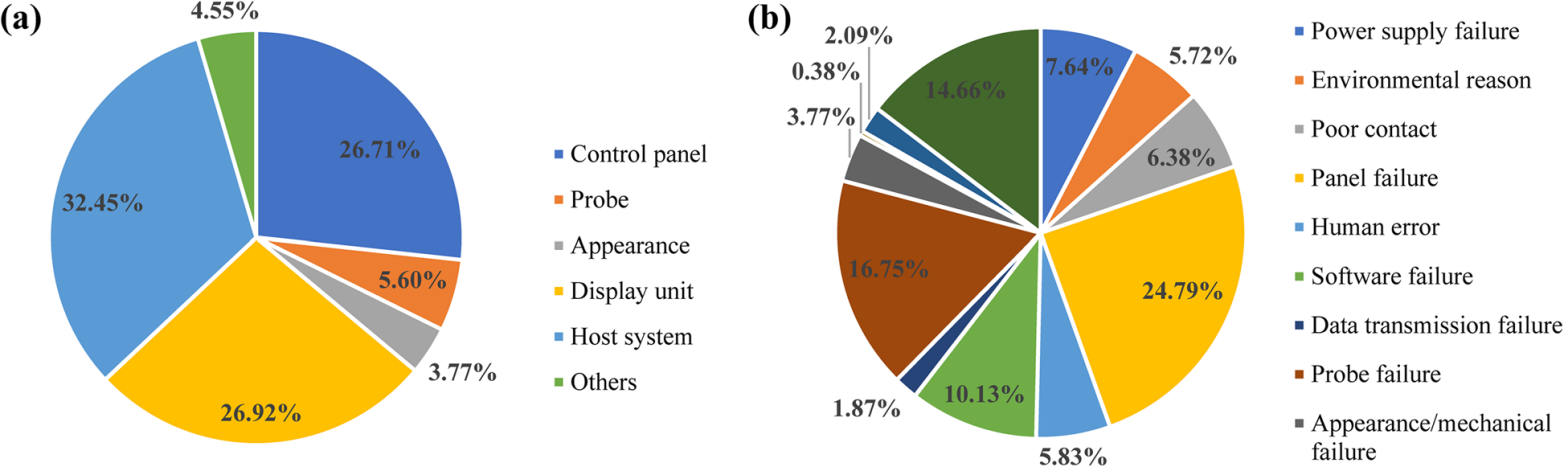
Percentages of different failure mode locations and failure cause categories. **a** Percentages of different failure mode locations of ultrasound devices. **b** Percentages of various failure cause categories of ultrasound devices

For the failure modes, the most common modes were control key malfunction (17.55%), unable to power on (11.69%) and unclear image (10.37%). As shown in Fig. 3a, the problems mainly occurred in the host system, display unit and control panel, with 32.5%, 26.9% and 26.7% of failures associated with these components, respectively. Regarding the failure causes, the most frequent ones were control key failures (16.18%), software system failures (10.13%), board failures (6.64%) and probe crystal failures (6.40%). As shown in Fig. 3b, of the major cause categories, panel failures (24.78%), probe failures (16.75%) and hardware processing unit failures (14.66%) were the three most frequent failure types. Additionally, 5.83% of failures were caused by human error, and 5.72% of failures were related to environmental factors.

**Fig. 4:**
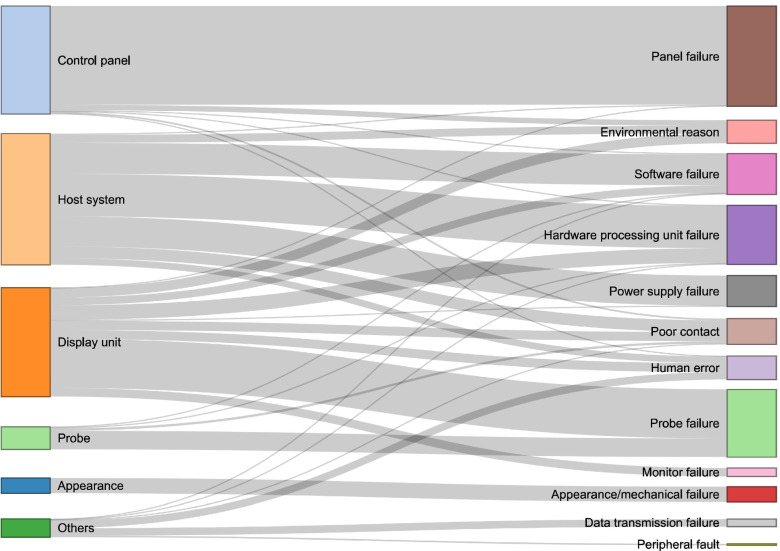
Correspondence between failure mode locations and major types of failure causes

According to the correspondence between every failure mode and cause, visual correlation analysis was conducted for the six kinds of failure mode locations and twelve major types of failure causes, as shown in Fig. 4. The main reason for the failure of the control panel was the panel itself. The main faults in the host system were related to the hardware processing unit, software and power supply. The specific causes of display unit system failure were complex and included probe failure, hardware processing unit failure and environmental conditions. In addition to probe failure, other probe issues were caused by poor contact, software failure and hardware processing unit failure. The failures generated in the appearance system were all mechanical faults. Other failures were mainly related to data transmission problems and human operator error.

### RPN of the failure mode

**Fig. 5:**
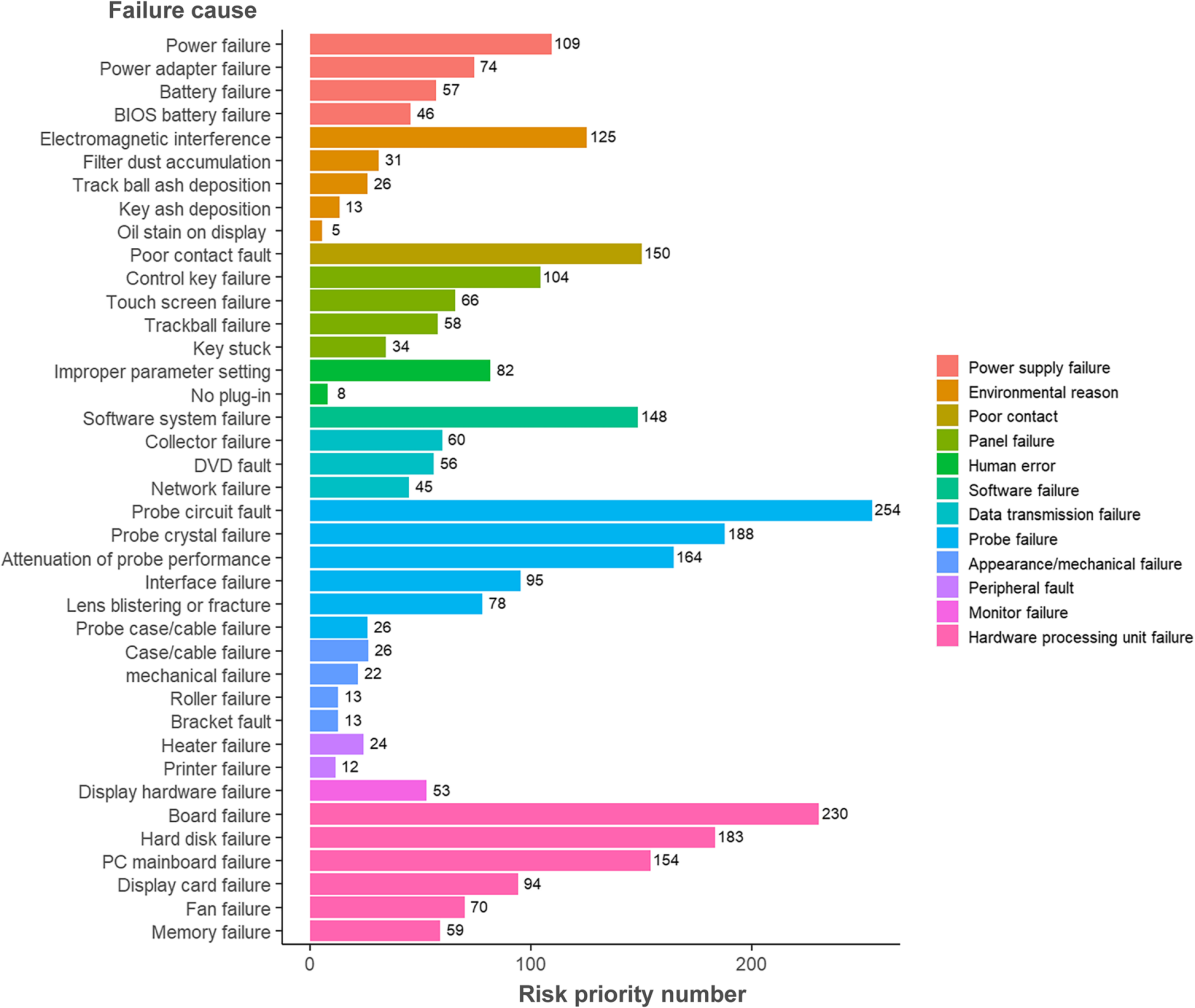
Risk priority numbers of failure causes

To quantitatively evaluate the risk of failure modes, the RPNs of different failure causes were determined first. The severity and detectability of the failure causes were scored using the Delphi expert consultation method. To assess the occurrence of failure, the proportion of failures in each category was used to represent the frequency. On the basis of severity, occurrence and detectability, the RPNs were calculated for the failure causes. As shown in Fig. 5, RPNs of 39 failure causes categorized in 12 types were obtained. Different failure cause types were distinguished by different colors. It could be seen that the RPNs of probe failure and hardware processing unit failure were the highest. For specific failure cause, probe circuit faults, board failure and probe crystal failure were the failure causes with the top three priority risk scores.

**Fig. 6:**
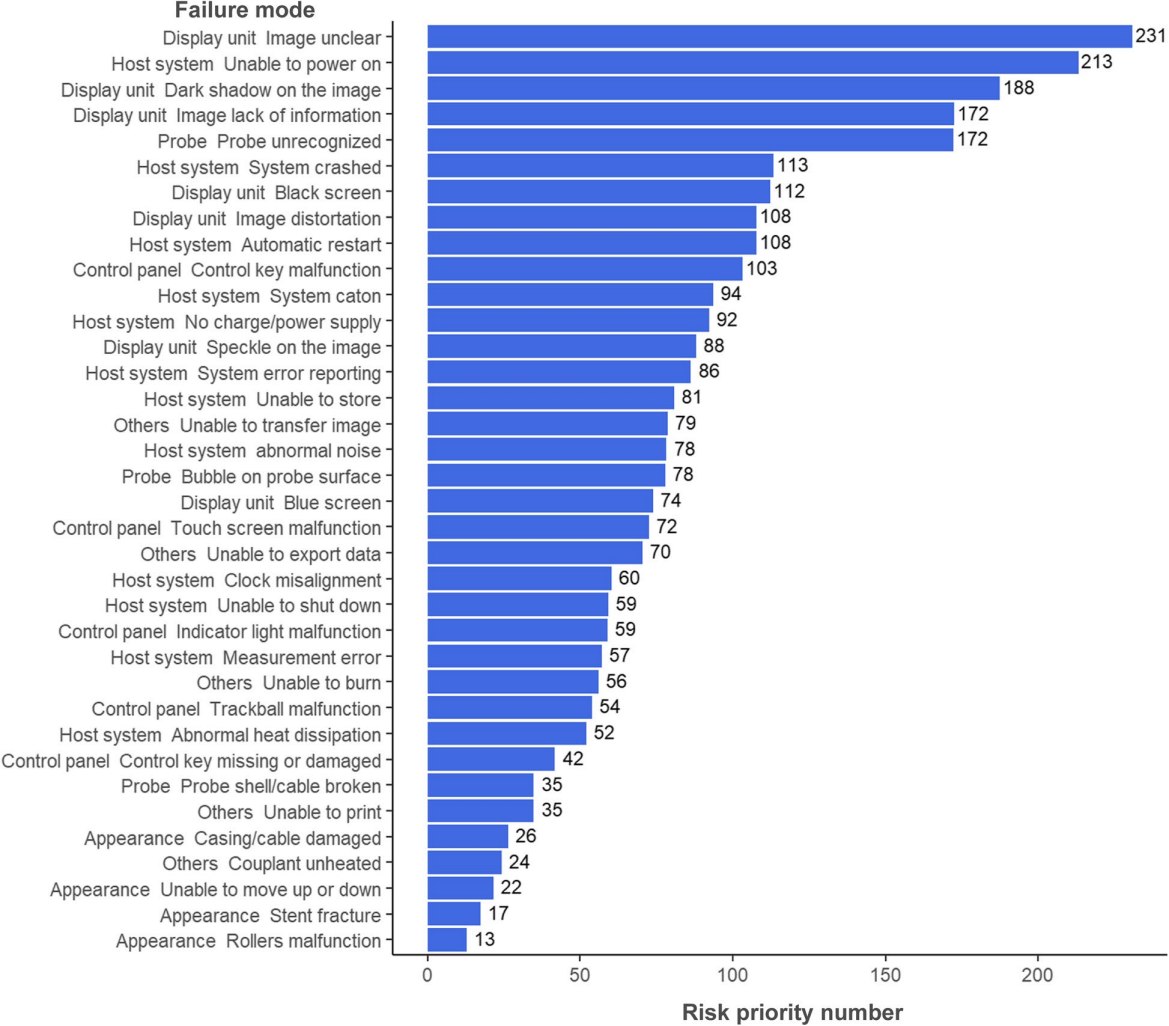
Risk priority numbers of failure modes

According to the calculation mode in the Methods section, the RPNs for 36 categories of failure modes were determined. As depicted in Fig. 6, unclear images, inability to power on and dark shadows on an image had the highest RPNs, which meant that such issues could potentially had the greatest impact on ultrasonic diagnostic procedures. Based on the failure mode severity calculation results, dark shadows on an image were the most crucial issue, with lack of information and abnormal noise also receiving high scores. Couplant heating and roller malfunction issues had the least severe impacts. With regard to detection difficulty, an unrecognized probe and lack of image information were the least common issues. Control key malfunction was the most frequently occurring failure type, which suggests that this kind of failure occurs frequently. However, the severity and detection difficulty of such failures were very low, which caused scant attention given to such issues.

**Fig. 7:**
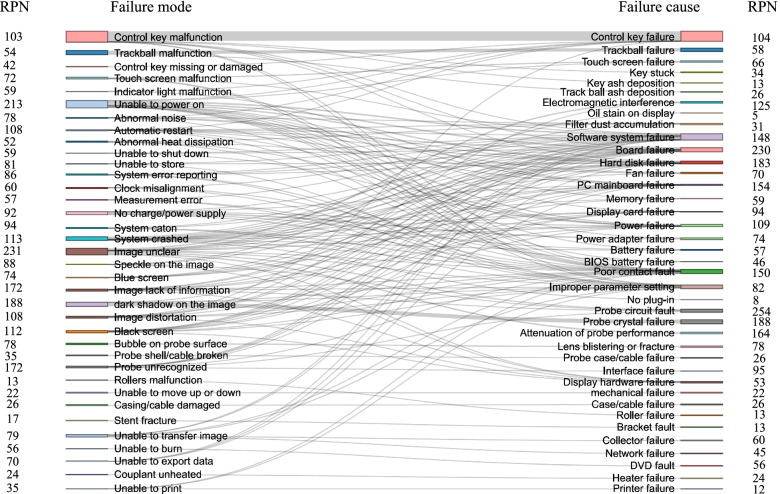
Correlation between RPN of failure modes and failure causes

Then, according to the correspondence diagram of the failure mode and failure cause, the RPNs of failure mode and causes were connected by the failure classification. As shown in Fig. 7, the RPN of the failure mode and failure cause were not exactly one-to-one correspondence, which was because that one failure mode might be caused by multiple failure causes. Only when the failure mode corresponded to one failure cause, their RPNs were the same. When a failure of ultrasound device occurred, this diagram could not only help physicians evaluate the risk of this failure mode, but also find out the most likely failure causes according to its path tracking, so as to take corresponding measures to solve the failure.

## Discussion

The higher RPN is, the greater the risk. The RPN can be used to assess the necessity of measures taken and which measure should be prioritized to reduce loss after a failure and improve the reliability of the system, thereby reducing the associated cost. On the basis of the results, control panel failure is the most frequent failure type. Unclear images are associated with the maximum severity and are the most difficult to detect. Overall, unclear images pose the greatest RPN, which means that such issues can potentially have the greatest impact on ultrasonic diagnostic procedures. Thus, for a failure mode with a high RPN, appropriate quality control measures should be implemented to reduce the effects of failures.

Unclear images, dark shadows on images and lack of image information, which all occur in the display unit, are mainly caused by probe crystal failure, the attenuation of probe performance and probe circuit failure. In addition, electromagnetic interference, improper parameter setting, software system failure, display hardware failure and other issues also cause abnormal displays. In view of the above problems, some measures should be regularly taken to reduce the risk of display unit failures including training users more frequently in how to hold the probe, keep the probe clean and wipe the coupling agent with soft paper after use and using dedicated power supplies for ultrasound devices to reduce the interference caused by other electrical devices.

Power failure, system crashes and automatic restarts are the three most serious failure modes in a host system. These issues are mainly caused by software system failure, board failure, hard disk failure and poor contact faults. The preventive strategies for such failures are as follows: reduce the use of portable storage devices to avoid software viruses, back up the system and reinstall the software whenever necessary, perform regular device maintenance to determine whether there are bad sectors in the hard disk, keep the device clean and reduce poor contacts.

Control key malfunction is the most frequent failure mode due to the large number of panel knobs, frequent use of devices, improper operation, accumulation of dust and other reasons. Unrecognized probe issues are mainly caused by probe circuit faults, poor contact faults and interface failures. Therefore, it is necessary to perform maintenance and keep the device clean in clinical practice.

The major highlights of this study included a quantitative assessment of the risks of different failure modes based on a large number of medical ultrasound devices at hospitals in multiple districts. Additionally, a FMECA method was adopted to determine the level of risk for different failure modes. Because one failure type may be caused by various factors, a visual failure mode tracing diagram was created, from which the classification of failures and the correlations between the failure modes and failure causes were identified. The RPN of each failure mode was obtained based on the RPN of the cause and the corresponding relationship between the phenomenon and cause.

A quantitative FMEA approach was utilized for linear accelerator quality assurance [22]. The SOD was assessed for potential risks. Few previous studies have investigated the risk associated with different types of failure modes of medical ultrasound devices. To analyze the failure mode of ultrasound device, researcher once categorized the failures of ultrasound devices were into 5 types based on 108 cases [16]. To the best of our knowledge, no previous research has analyzed such a large amount of ultrasound device failure data or the correlations between failure modes and causes. Additionally, quantitative assessments of the potential impacts of failure modes in various categories have never been conducted. Given that the final effect of failure is directly related to the cause, this FMECA risk assessment approach is more accurate than traditional methods.

Through quantitative scoring of each failure mode, it was found that the three failure modes with the highest RPN value were unclear image, unable to power on and dark shadow on the image. Ultrasound equipment users could make corresponding plans according to the risk score of failure mode to avoid adverse events. In addition, according to the corresponding diagram of failure mode and cause obtained from the analysis, it was helpful for the physicians and clinical engineers to find the cause of the failure, which can reduce the impact on the clinical diagnostic process, the potential risks to patient safety and economic loss. In addition, the frequency of failures with high-risk values can be used as an index to evaluate the reliability of ultrasound devices, thus providing direction regarding technical enhancements to manufacturers and improving device selection and purchasing methods.

Our study had some limitations. It is worth noting that due to the diversity of ultrasound device maintenance service providers, it was impossible to guarantee coverage for all failure records for such a large number of ultrasound devices. We collected as many samples as possible in a variety of areas. Second, although we performed a quantitative evaluation of the risks of different failure modes, potential safety hazards for patients and physicians should be further discussed.

## Conclusions

This study was conducted to quantitatively evaluate the risk of different failure modes of ultrasound devices based on a FMECA approach. Through a retrospective analysis of a large sample of maintenance records, the corresponding relationships between the failure modes and failure causes were established. By applying the analysis calculation model, the RPN for each failure mode was finally obtained. The failure mode of image unclear had the maximum risk priority number, with the high frequency of occurrence, severity and detectability value. According to the risk level of different failure modes, the device administrator can make corresponding preventive plans. And the correspondence analysis of failure modes and causes can be conducive to determining the cause of failure, thus reducing the occurrence of adverse events and economic losses.

Future research should evaluate the threshold of the RPN of the failure mode of ultrasound device. When this threshold is exceeded, the device needs to be stopped immediately for repair or replacement, or further measures should be taken to protect the patient’s safety. Moreover, the failure risk rating of ultrasound device applied in different fields should not be the same. For example, the severity of transesophageal cardiac probe failure is much higher than that of conventional cardiac probe during the disease diagnosis. The safety risk caused by ultrasound failure in surgical intervention is much greater that in routine physical examination. Therefore, research on the quantitative assessment of ultrasonic failure risk in specific application fields will be more targeted to reduce the safety risk.

## Data Availability

The datasets used during the current study are available from the corresponding author on reasonable request.

## Abbreviation

RPN: Risk priority number; FMECA, failure mode, effect and criticality analysis
